# Comparison of the Clinical and Metabolic Characteristics of Patients With Acute Coronary Syndromes Between the Pre- and Post-lockdown Periods

**DOI:** 10.7759/cureus.46754

**Published:** 2023-10-09

**Authors:** Tommaso Capobianco, Walther Iannotti, Riccardo Agostini, Luca Persiani, Marco Chiostri, Giorgio Iacopo Baldereschi, Carlo Di Mario, Francesco Meucci, Renato Valenti, Emanuele Cecchi

**Affiliations:** 1 Department of Cardiac, Thoracic, and Vascular Medicine, Azienda Ospedaliero Universitaria Careggi, Florence, ITA

**Keywords:** metabolic alterations, young patients, acute coronary syndromes, covid-19, lockdown

## Abstract

Introduction: In 2020, the SARS-CoV-2 pandemic outbreak required restrictive measures to limit the spread of the virus. This study aimed to assess how changes in dietary habits and lifestyle associated with such measures have affected the characteristics of patients with acute coronary syndromes (ACS) in the post-lockdown period. In particular, we evaluated if the incidence of ACS was higher in younger patients, who were more negatively affected by lockdown measures.

Methods: We analysed 609 ACS patients and compared the clinical, laboratory, and angiographic characteristics of those admitted six months before lockdown (n = 312) and those admitted in the same six-month period after lockdown. Moreover, we compared several anthropometric and laboratory data between pre- and post-lockdown in younger (≤55 years old) and older patients.

Results: The incidence of ACS in young adults (≤55 years) was significantly higher in the post- vs. pre-lockdown period (17.5% vs. 10.9%, p = 0.019). A trend to a higher percentage of ST-elevation myocardial infarction (STEMI) was observed in the post-lockdown period together with a significantly lower incidence of non-STEMI (p = 0.033). Moreover, in the post-lockdown period, we observed in younger patients a significant increase in weight, body mass index, admission glycaemia, and triglycerides while in older patients, these parameters were significantly reduced.

Conclusion: The lockdown may have negatively affected cardiovascular risk, thus increasing the incidence of ACS, particularly in younger patients who probably underwent more relevant lifestyle changes, with several consequent anthropometric and metabolic alterations. Such evidence should be considered to take preventive measures in case a new state of emergency occurs.

## Introduction

In December 2019, following the reporting of some acute respiratory syndrome outbreaks, a new coronavirus, subsequently named SARS-CoV-2, was isolated in Wuhan, China [1]. Due to its high contagiousness, the virus had such a rapid spread worldwide that the World Health Organization declared it a pandemic in March 2020.

Italy was the first Western country to deal with the socio-sanitary consequences arising from SARS-CoV-2. On the 9th of March 2020, due to the outbreak of the pandemic, a national lockdown was imposed, and it established a ban on leaving dwellings unless proven work, family, or health needs, and also the closure of educational, commercial, and recreational activities.

These measures led to a drastic change in lifestyle and in particular in the most active share of the population, i.e., young adults. Social isolation eased those behaviours that are notoriously associated with increased cardiovascular risk, such as unhealthy eating habits, sedentary lifestyles, and unnecessary habits [2-4].

From a nutritional point of view, a quantitative increase in the consumption of food exceeding the energy requirement was observed. Sedentary and other aspects just described associated with the lockdown period increased cardiometabolic risk factors, such as hyperglycaemia and hyperinsulinemia, leading to hyperproduction of pro-inflammatory and pro-atherosclerotic cytokines [5].

These aspects had a different impact on the population. The most affected by these lifestyle changes during the lockdown were certainly the young adults who were the most active in the pre-lockdown period. Several studies have shown how social isolation has prevented this population group in particular from carrying out the life to which they were accustomed by reducing the possibility of physical activity and engaging more time in sedentary activities (e.g., smart working, watching TV, and using social networks) and changing their eating habits [6-8].

Based on this evidence, we aimed at comparing clinical and metabolic characteristics in acute coronary syndrome (ACS) patients between pre- and post-lockdown periods.

## Materials and methods

Our study is a retrospective observational analysis, and data have been collected from the Department of Cardiac, Thoracic, and Vascular Medicine of the Azienda Ospedaliero-Universitaria Careggi in Florence, a tertiary hub centre with on-site cath-lab availability 24 hours per day/seven days per week/365 days per year. We have chosen to compare the period of six months immediately before the lockdown in Italy (9 September 2019 to 9 March 2020) with the same six-month period after the lockdown (9 September 2020 to 9 March 2021), during which we have selected all consecutive patients admitted with a diagnosis of ACS treated with percutaneous angioplasty. The only inclusion criterion was an age higher than 18 years old. ACS patients treated with medical therapy or coronary artery bypass graft were excluded from the study. Diagnosis of ACS includes ST-elevation myocardial infarction (STEMI), non-STEMI (NSTEMI), or unstable angina (in accordance with the latest European Society of Cardiology (ESC) guidelines) [9-11]. First, we compared the clinical characteristics of all patients between the pre-lockdown and post-lockdown period. Our primary endpoint was to evaluate if there was any difference in the incidence and type of ACS in the overall group of patients and in the subgroup of patients less than 55 years old in the post-lockdown compared to the pre-lockdown period. Moreover, we evaluated, as secondary endpoints, differences in several angiographic characteristics between the pre- and post-lockdown period as well as in several anthropometric and laboratory parameters associated with a metabolic profile between the pre- and post-lockdown period in patients ≤ 55 years old and in those > 55 years old. All patients gave their informed consent for the enrolment in this study. The study protocol has been drawn in accordance with the ethical guidelines of the 1975 Declaration of Helsinki and was approved by the local ethics committee.

Statistical analysis

Statistical analysis was performed using SPSS Statistics software (version 28.0; IBM Corp., Armonk, NY). Regarding statistical calculations for dichotomous variables (categorical variables), we calculated the absolute number and percentage of the variables analysed in the two periods and then compared differences between them by means of the chi-square test. Instead, continuous variables were expressed as median and range or mean ± standard deviation when appropriate and differences among them were calculated by means of the Mann-Whitney test. Statistical significance was expressed by a p-value < 0.05.

## Results

In the analysed periods, we studied 609 patients with a diagnosis of ACS admitted to our centre (University Hospital Careggi in Florence); 312 (223 males and 89 females) in the pre-lockdown period and 297 patients (223 males and 74 females) in the post-lockdown period. Characteristics of the two patient groups are reported in Table 1.

**Table 1:** Comparison between clinical characteristics of acute coronary syndrome patients between the pre- and the post-lockdown period. CAD = coronary artery disease; ACS = acute coronary syndrome; PCI = percutaneous coronary intervention; CABG = coronary artery bypass graft.

Patients characteristics	Pre-lockdown (N = 312)	Post-lockdown (N = 297)	P-value
Age, median (range)	73 (39-93)	71 (27-95)	0.181
Patients ≤ 55 years old, n (%)	34 (10.9)	52 (17.5)	0.019
Males, n (%)	223 (71.5)	223 (75.1)	0.315
Arterial hypertension, n (%)	219 (70.2)	187 (63.0)	0.059
Dyslipidemia, n (%)	158 (50.6)	124 (41.8)	0.028
Diabetes mellitus, n (%)	73 (23.4)	76 (25.6)	0.529
Active smokers, n (%)	86 (27.6)	68 (22.9)	0.185
Former smokers, n (%)	87 (27.9)	71 (23.9)	0.263
Chronic obstructive pulmonary disease, n (%)	18 (5.8)	24 (8.1)	0.260
Family history of CAD, n (%)	68 (21.8)	54 (18.2)	0.265
Peripheral obliterative arteriopathy, n (%)	17 (5.4)	12 (4.0)	0.415
Carotid artery disease, n (%)	23 (7.4)	21 (7.1)	0.886
Chronic kidney disease, n (%)	11 (3.5)	26 (8.8)	0.007
Previous ACS, n (%)	84 (26.9)	80 (26.9)	0.997
Previous PCI, n (%)	87 (27.8)	83 (27.9)	0.987
Previous CABG, n (%)	6 (1.9)	13 (4.4)	0.082
Previous SARS-CoV-2 infection, n (%)	0 (0)	2 (0.7)	NA

We observed that the median age of patients hospitalized in the post-lockdown period was lower, though not significant, with respect to those in the pre-lockdown period (71 years vs. 73 years). The median age and the other characteristics of younger and older patients in analysed periods are reported in Table 2. In relation to our goal, we have found that in the post-lockdown period, the number of patients ≤ 55 years old diagnosed with ACS was significantly higher. In fact, in the pre-lockdown period, the patients ≤ 55 years old with ACS were 10.9% (34 patients out of 312 total), while in the post-lockdown period, they were 17.5% (52 patients out of 297 total) (p-value = 0.019) (Table 1). Considering the overall population studied, patients in the pre-lockdown period had a significantly higher prevalence of dyslipidemia while chronic kidney disease was significantly more frequent in patients in the post-lockdown period. Considering the type of ACS, we observed in the post- vs. pre-lockdown period a significantly lower incidence of NSTEMI (p = 0.033) and a non-significant trend to a higher incidence of STEMI but no significant variation in the type of ACS in the subgroup of patients ≤ 55 years old (Table 2).

**Table 2:** Comparison of acute coronary syndromes characteristics, angiographic parameters, biomarkers of myocardial damage, and ejection fraction between the pre- and post-lockdown period. STEMI = ST-elevation myocardial infarction; NSTEMI = non-ST-elevation myocardial infarction; PCI = percutaneous coronary intervention; CK-MB = creatine kinase-myocardial band; Tn-T hs = troponin T, high sensitivity.

ACS characteristics	Pre-lockdown	Post-lockdown	P-value
	N = 312	N = 297	
STEMI, n (%)	120 (38.5)	134 (45.1)	0.096
NSTEMI, n (%)	140 (44.9)	108 (36.3)	0.033
Unstable angina, n (%)	52 (16.6)	55 (18.5)	0.548
STEMI in patients ≤ 55 years, n (%)	22 (64.7)	34 (65.4)	0.949
NSTEMI in patients ≤ 55 years, n (%)	9 (26.5)	10 (19.2)	0.344
Unstable angina in patients ≤ 55 years, n (%)	3 (8.8)	8 (15.4)	0.513
Single vessel disease, n (%)	133 (42.6)	125 (42.1)	0.893
Two vessel disease, n (%)	113 (36.2)	117 (39.4)	0.419
Three vessel disease, n (%)	66 (21.2)	55 (18.5)	0.415
Left main involvement, n (%)	35 (11.2)	39 (13.1)	0.470
Intrastent restenosis, n (%)	27 (8.7)	12 (4.0)	0.020
Mean number of stents implanted	2.04 ± 1.39	1.77 ± 1.11	0.031
Mean number of vessels treated per PCI	1.75 ± 0.89	1.61 ± 0.89	0.053
Median peak CK-MB values (ng/mL)	18.8 (0.9-817.0)	13.8 (0.8-600.0)	0.061
Median peak Tn-T hs values (pg/mL)	700 (6-60100)	677 (7-100000)	0.409
Median ejection fraction at the admission, n (%)	55 (10-68)	50 (10-65)	0.524
Death during hospitalization, n (%)	9 (2.9)	12 (4.0)	0.435
Median length of stay, n (days)	8.4 ± 9.0	7.5 ± 7.3	0.028

Moreover, we did not find any difference in the severity of coronary artery disease (single-vessel, two-vessel, three-vessel, left main involvement) (Table 2) between the two groups analysed.

Among the angiographic characteristics of patients studied in the overall population in the pre-lockdown period, a higher prevalence of intrastent restenosis (8.7% in the pre-lockdown vs. 4.0% in the post-lockdown, p = 0.02) was observed, together with a significantly higher number of stents implanted (2.04 ± 1.39 vs. 1.77 ± 1.11, p = 0.031). Moreover, also median length of hospital stay resulted significantly higher in the pre- vs. post-lockdown period (p = 0.028).

**Figure 1:**
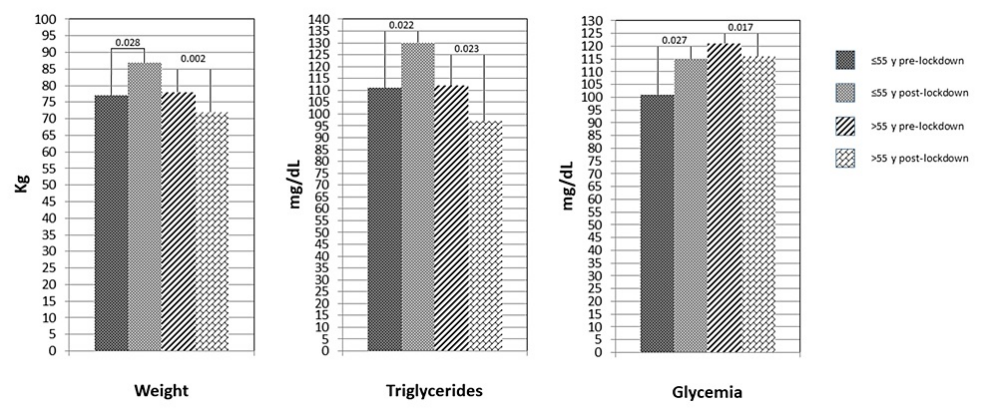
Opposite behaviour in the variations of anthropometric and metabolic variables from the pre-lockdown to the post-lockdown period between younger (≤55 years old) and older patients. y = years.

Finally, considering the main anthropometric and laboratory variables associated with the metabolic profile, we observed a statistically significant increase in body weight and body mass index (BMI) together with a significant increase in triglycerides and admission glycaemia in the post- vs. pre-lockdown period in patients ≤ 55 years old (Table 3 and Figure 1); conversely, in patients over 55 years, a significant reduction in body weight, BMI, triglycerides, and admission glycaemia was observed (Table 3 and Figure 1).

**Table 3:** Comparison of several anthropometric, laboratory, and echocardiographic parameters between the pre- and post-lockdown period in young patients (≤ 55 years old) and in older ones. BMI = body mass index; HDL = high-density lipoprotein; LDL = low-density lipoprotein; eGFR = estimated glomerular filtration rate; CK-MB = creatine kinase-myocardial band; Tn-T hs = troponin T, high sensitivity. Values are expressed as median and range.

Variables	Patients	≤55 years		Patients	>55 years	
	Pre-lockdown	Post-lockdown	P-value	Pre-lockdown	Post-lockdown	P-value
Age (years)	51 (39-55)	51 (27-55)	0.766	74 (56-93)	74 (56-95)	0.910
Weight (kg)	77 (47-110)	87 (44-108)	0.028	78 (42-113)	72 (48-127)	0.002
Height (cm)	172 (156-186)	174 (160-186)	0.784	170 (145-198)	170 (150-190)	0.381
BMI (kg/m2)	25.3 (19.3-39.4)	27.8 (16.8-41.0)	0.018	26.6 (16.0-36.2)	25.2 (18.3-41.5)	0.003
Total cholesterol (mg/dL)	178 (57-258)	170 (101-1148)	0.521	153 (55-418)	155 (77-321)	0.865
HDL cholesterol (mg/dL)	38 (28-60)	39 (21-97)	0.715	42 (23-134)	45 (25-117)	0.017
LDL cholesterol (mg/dL)	133 (20-191)	121 (42-209)	0.374	95 (22-353)	95 (28-239)	0.495
Triglyceridemia (mg/dL)	111 (31-266)	130 (55-311)	0.022	112 (31-295)	97 (43-393)	0.023
Haemoglobin (g/dL)	14.6 (10-17)	14.7 (8-17)	0.769	13.7 (8-20)	13.5 (8-19)	0.182
Serum creatinine (mg/dL)	0.89 (0.54-1.53)	0.84 (0.54-9.47)	0.264	1.01 (0.49-9.00)	0.94 (0.43-10.06)	0.153
eGFR (mL/min/1.73m2)	96 (50-139)	98 (5-171)	0.367	72 (5-147)	77 (5-181)	0.104
Glycaemia (mg/dL)	101 (80-342)	115 (84-365)	0.027	121 (42-607)	116 (39-963)	0.017
Peak Tn-T hs (pg/mL)	1901 (11-38324)	805 (10-22578)	0.184	632 (6-60100)	612 (7-100000)	0.602
Peak CK-MB (ng/mL)	61 (1.0-809.0)	22.0 (1.1-600.0)	0.217	16.8 (0.9-817.0)	12.7 (0.8-600.0)	0.096
Ejection fraction (%)	55 (10-68)	53 (25-65)	0.490	55 (10-68)	50 (10-65)	0.450

## Discussion

Our study focused on analysing the effects of the lockdown period due to the SARS-CoV-2 pandemic on clinical characteristics of patients presenting with ACS comparing those admitted six months before lockdown with those admitted in the same six-month time frame after lockdown. In fact, during that period, the conditions for an unprecedented increase in cardiovascular risk factors were created.

First, as the type of ACS is concerned, we observed a significantly higher incidence of NSTEMI in the pre- vs. post-lockdown period and a trend towards a higher percentage of STEMI in the post-lockdown period. This might in part reflect, after lockdown, the fear of patients to go to the hospitals, except in case of persisting or evolving symptoms such as in the case of STEMI [12].

Moreover, since young adults during lockdown underwent the greatest lifestyle changes, we wondered if these changes had an impact on the incidence of ACS in this age group.

The main result of our study is that the incidence of ACS was significantly higher in patients younger than 55 years after the lockdown period with respect to the same six-month time frame before the lockdown. This result confirms our initial hypothesis and is in line with the increase in sedentary lifestyle, the reduction in physical activity, and an improper diet that were observed during the lockdown, and which mainly affected the most active part of the population.

Home confinement, the introduction of smart working, the closure of fitness centres, and the lack of motivation contributed to an increase in daily sedentary time and, at the same time, a reduction in physical activity compared to the pre-lockdown period [13-16]. From a nutritional point of view, a quantitative increase in the consumption of food exceeding the energy requirement was observed.

Furthermore, worse quality of diet was highlighted with reduced adherence to the Mediterranean diet, in particular in European countries, with a shift to diets rich in carbohydrates and lipids and a net increase in the intake of junk food, ultra-processed foods, snacks, and long-life canned foods [6,17].

These factors, obviously, led to weight gain associated also with an increase in triglycerides and glycaemia in the younger population [2,7,17,18]. In a French study on 536 patients, an increase in the median body weight of 2 kg in the post-lockdown period was observed [19]. In agreement with our hypothesis and evidence in the literature, we observed in younger patients after lockdown a significant increase in median body weight and BMI as well as median values of glycaemia and triglycerides.

Alcohol consumption also increased during the lockdown period [2]. About a quarter of alcohol users reported an increase in their alcohol intake [20].

On the other hand, there was a statistically significant reduction in body weight, BMI, glycaemia, and triglycerides in the elderly population. Accordingly, in an Italian study [21], the elderly population (>60 years) showed, in contrast to the general population, a reduction in body weight, likely associated with malnutrition.

With regard to cigarette smoking, although some studies have observed an increase in the number of smoked cigarettes [3,13,22], other studies have not observed this trend making the data available quite inconsistent, so it is presumed that the lockdown would only have a mild effect on tobacco habit [2]. In our analysis, we did not show a significant difference in cigarette consumption in the post-lockdown compared to the pre-lockdown period.

Our results are coherent with a recent meta-analysis that analysed five systematic reviews and six prospective trials, reporting the effect of a sedentary lifestyle on chronic cardiovascular diseases was observed by analysing the possible pathophysiological mechanisms [5]; it was highlighted how a sedentary lifestyle and the prolonged sitting position causes dysregulation of several hormones that control appetite and caloric expenditure such as leptin and ghrelin, alters the adiponectin signalling, modifies the morphology of the adipose tissue, promotes thyroid dysregulation, and all these alterations associated with a greater caloric intake favours the onset of overweight and obesity. Moreover, a sedentary lifestyle also favours the onset of arterial hypertension, dyslipidemia, and insulin resistance and could promote plaque instability causing an increased expression of cytokines, interleukins, and reactive oxygen species with a consequent increased risk of acute cardiovascular events. Several studies have confirmed the association between inflammation and instability of the atherosclerotic plaque [23-25], favouring ACS occurrence.

Finally, no significant differences were found in the severity of coronary heart disease and in acute clinical complications either by comparing patients ≤55 years old or by comparing the overall group of ACS patients in the pre-lockdown and post-lockdown period. On coronary angiography, one significant difference found was the presence of a higher prevalence of intrastent restenosis in the general population of the pre-lockdown period, which could be in part ascribed to the higher prevalence of younger patients in the post-lockdown period as well as to a higher prevalence of NSTEMI respect to STEMI in the pre-lockdown period, since restenosis is more frequently observed in NSTEMI patients. Accordingly, a significantly higher number of stents were implanted during percutaneous coronary intervention in the pre-lockdown period, with a higher prevalence observed in older patients, which are often characterized by longer and more complex lesions requiring multiple stent implantations.

Interestingly, our study showed how the lockdown period related to the SARS-CoV-2 pandemic had a heterogeneous impact on cardiovascular risk factors. In younger patients with ACS during the pandemic period, we observed a worsening of the glycolipidic metabolic profile and an increase in body weight. This is attributable to a reduction in attention to a correct lifestyle. Instead, it would seem that home confinement had a minor influence on other classic risk factors, such as tobacco exposure and systemic arterial hypertension.

These findings, in agreement with the data present in the literature, must be strongly taken into consideration in the event of a new pandemic or similar emergency situations, which require restrictive measures, occur. In such contexts, in fact, it will be fundamental, in terms of cardiovascular prevention, to engage with the education of the general population either in correct nutrition or in a program of minimal regular physical activity, in particular in younger subjects. The importance of cardiovascular disease prevention during the COVID-19 pandemic has been recently emphasized [26,27].

Limitations

Interpretation of data from this study must take into account some limitations. First of all, these results are derived from a retrospective analysis of single-centre data. Secondly, we have selected the cut-off of 55 years to identify young patients, although various cut-offs have been proposed in the literature, and there is no univocal agreement on which age is the most appropriate to identify premature ACS patients.

## Conclusions

The SARS-CoV-2 pandemic and the lockdown caused several lifestyle changes across the world population. The young adult population suffered most of the consequences of these changes. Increased sedentary time, reduced activity time, and improper diet may increase cardiovascular risk and the incidence of acute coronary events in young adults, as observed in our centre. Our study could be a starting point for further studies and suggests the need for appropriate strategies to prevent drastic lifestyle changes in case of future pandemics.
